# Predicting Cloud‐To‐Ground Lightning in the Western United States From the Large‐Scale Environment Using Explainable Neural Networks

**DOI:** 10.1029/2024JD042147

**Published:** 2024-11-22

**Authors:** Dmitri A. Kalashnikov, Frances V. Davenport, Zachary M. Labe, Paul C. Loikith, John T. Abatzoglou, Deepti Singh

**Affiliations:** ^1^ School of the Environment Washington State University Vancouver WA USA; ^2^ Sierra Nevada Research Institute University of California, Merced Merced CA USA; ^3^ Civil and Environmental Engineering Colorado State University Fort Collins CO USA; ^4^ Atmospheric and Oceanic Sciences Program Princeton University Princeton NJ USA; ^5^ NOAA/Geophysical Fluid Dynamics Laboratory Princeton University Princeton NJ USA; ^6^ Department of Geography Portland State University Portland OR USA; ^7^ Management of Complex Systems Department University of California, Merced Merced CA USA

**Keywords:** lightning, wildfires, neural network, machine learning, prediction

## Abstract

Lightning is a major source of wildfire ignition in the western United States (WUS). We build and train convolutional neural networks (CNNs) to predict the occurrence of cloud‐to‐ground (CG) lightning across the WUS during June–September from the spatial patterns of seven large‐scale meteorological variables from reanalysis (1995–2022). Individually trained CNN models at each 1° × 1° grid cell (*n* = 285 CNNs) show high skill at predicting CG lightning days across the WUS (median AUC = 0.8) and perform best in parts of the interior Southwest where summertime CG lightning is most common. Further, interannual correlation between observed and predicted CG lightning days is high (median *r* = 0.87), demonstrating that locally trained CNNs realistically capture year‐to‐year variation in CG lightning activity across the WUS. We then use layer‐wise relevance propagation (LRP) to investigate the relevance of predictor variables to successful CG lightning prediction in each grid cell. Using maximum LRP values, our results show that two thermodynamic variables—ratio of surface moist static energy to free‐tropospheric saturation moist static energy, and the 700–500 hPa lapse rate—are the most relevant CG lightning predictors for 93%–96% of CNNs depending on the LRP variant used. As lightning is not directly simulated by global climate models, these CNNs could be used to parameterize CG lightning in climate models to assess changes in future CG lightning occurrence with projected climate change. Understanding changes in CG lightning risk and consequently lightning‐caused wildfire risk across the WUS could inform fire management, planning, and disaster preparedness.

## Key Points

- Convolutional Neural Networks (CNNs) trained on seven meteorological variables show skill at predicting CG lightning days in the WUS, performing best in the Southwest
- Year‐to‐year variation in lightning activity is robustly captured by the CNNs (domain median *r* = 0.87)
- MSE ratio and 700–500 hPa lapse rate are the top CG lightning predictors in most grid cells

## 1 Introduction

In the western United States (WUS), summertime cloud‐to‐ground (CG) lightning is an important wildfire ignition source when occurring with limited precipitation (i.e., “dry lightning”). Although humans are responsible for most wildfire ignitions, lightning‐caused fires account for the majority of burned area across this region (Abatzoglou et al., 2016; Balch et al., 2017; Brey et al., 2018; Janssen et al., 2023; Kalashnikov, Abatzoglou, et al., 2022; Komarek, 1967). Smoke from such fires can also have far‐reaching air quality and human health impacts. For example, in August 2020, smoke from wildfires ignited by a widespread dry lightning outbreak in California resulted in extreme levels of air pollution across more than two‐thirds of the WUS (Kalashnikov, Schnell, et al., 2022) and contributed to high human mortality and morbidity in multiple states (Rosenthal et al., 2022; Zhou et al., 2021). Projected warming in the WUS is likely to increase burned area from wildfires—including those ignited by lightning—due to drier fuels (Barros et al., 2021; Li et al., 2020; Pérez‐Invernón et al., 2023). Recent studies have also projected increases in lightning occurrence in the WUS (Finney et al., 2018; Janssen et al., 2023; Pérez‐Invernón et al., 2023), lending urgency to understanding and anticipating societal impacts with continued warming.

Complicating future projection efforts is the fact that lightning cannot be directly simulated at the typically coarse spatial resolutions of Global Climate Models (GCMs) because of their inability to simulate the fine‐scale physical processes associated with lightning activity. Previous studies of lightning using atmospheric reanalyzes and GCMs have relied on convective parameterizations utilizing cloud top height (Janssen et al., 2023; Krause et al., 2014; Pérez‐Invernón et al., 2023; Price & Rind, 1992), cloud droplet concentration (Michalon et al., 1999), cold cloud depth (Yoshida et al., 2009), convective ice flux (Finney et al., 2014, 2018; Janssen et al., 2023), cloud ice fraction (Han et al., 2021), convective precipitation and mass flux (Allen & Pickering, 2002; Magi, 2015), the product of convective available potential energy and precipitation rate (Chen et al., 2021; Romps et al., 2014), and other combinations of variables that are physically relevant for in‐cloud charge separation and lightning production (See Clark et al. (2017) and Etten‐Bohm et al. (2021) for a comprehensive summary of lightning parameterizations). However, substantial disagreement in lightning projections can arise when using different parameterizations, and the best approach is not yet clear (Clark et al., 2017; Romps, 2019; Tost et al., 2007). Further, there are inherent uncertainties in representing convective cloud properties in GCMs (Etten‐Bohm et al., 2021).

Due to these limitations, recent studies have developed relatively simple lightning parameterizations using large‐scale dynamic and thermodynamic meteorological variables, which are directly simulated by GCMs. For example, Etten‐Bohm et al. (2021) used logistic regression to predict lightning occurrence in the global tropics and subtropics from a set of dynamic and thermodynamic variables in reanalysis, with the intention of applying this statistical parameterization to GCMs. Similarly, Liu et al. (2022) used Random Forest models trained on a set of four meteorological variables to predict the global occurrence of thunderstorms. Bates et al. (2018) tested six statistical modeling approaches for predicting lightning days in Australia using a comprehensive set of 31 large‐scale meteorological variables and found that logistic regression performed best. Other studies have used multiple linear regression to predict lightning flash density from meteorological variables that broadly describe the convective state of the atmosphere (Stolz et al., 2017; Veraverbeke et al., 2017). It is worth noting that neural networks have been underutilized in lightning parameterization efforts in reanalyses and GCMs, with limited examples trained over relatively short time periods (e.g., Cheng et al., 2024).

We use multivariate CNNs trained on large‐scale meteorological variables over a 28‐year historical period (1995–2022) to parameterize lightning activity in the WUS and evaluate whether CNNs offer advantages over a traditional statistical classifier. CNNs are a type of supervised deep learning image classification (Goodfellow et al., 2016; LeCun et al., 2015) and have shown promising potential in atmospheric and climate science at capturing complex and nonlinear physical relationships (Baño‐Medina et al., 2021; Chattopadhyay et al., 2020; Dagon et al., 2022; Davenport & Diffenbaugh, 2021; Lagerquist et al., 2019; Molina et al., 2021; Trok et al., 2023). Another strength of CNNs is their inherent ability to differentiate spatial features as the input fields are two‐dimensional, which can facilitate robust learning of important atmospheric features for a given prediction task (Baño‐Medina et al., 2021; Molina et al., 2021). Importantly, “eXplainable Artificial Intelligence” (XAI), a term encompassing the growing ecosystem of machine learning visualization methods, can disentangle the contribution of different variables and their spatial features to predictions, thus enabling physical insights into the governing processes (Davenport & Diffenbaugh, 2021; Ebert‐Uphoff & Hilburn, 2020; Labe & Barnes, 2021; Mamalakis, Barnes, & Ebert‐Uphoff, 2022; Mamalakis, Ebert‐Uphoff, & Barnes, 2022; Mamalakis et al., 2023; McGovern et al., 2019; Molnar et al., 2022; Rudin, 2019; Toms et al., 2020). Here, we use an XAI technique known as layer‐wise relevance propagation (LRP; Bach et al., 2015) to investigate the relative importance of predictor variables to successful CG lightning prediction across the WUS.

In this study, we develop, train, and test individual CNNs to predict the daily occurrence of CG lightning at each 1° × 1° grid cell in the WUS (for a total of 285 CNNs) based on the spatial fields of seven large‐scale meteorological variables. WUS thunderstorms are relatively rare and understudied compared to severe thunderstorms that commonly affect the central and eastern US, particularly outside areas of the Southwest that are directly affected by the North American monsoon (Adams & Comrie, 1997; Barlow et al., 1998). Consequently, there is a lack of lightning parameterizations developed specifically for this region. One exception is the recent work by Cheng et al. (2024), who used a neural network to predict lightning flash density in the WUS. We build on this work by using CNNs trained at individual grid cells using locally centered meteorological fields that we demonstrate present substantial improvements in prediction skill. By developing individual CNNs at each grid cell and predicting CG lightning at the daily scale, our approach offers two primary advantages over traditional lightning parameterization methods. *First*, our targeted approach provides refined spatial and temporal resolution compared to parameterization methods that assessed bulk lightning activity at national to global scales and at monthly to annual aggregation (Allen & Pickering, 2002; Cheng et al., 2024; Clark et al., 2017; Finney et al., 2016, 2018; Magi, 2015; Price & Rind, 1992; Romps, 2019; Romps et al., 2014). Our development of predictor models at each grid cell is better suited for the WUS, since lightning climatology and associated meteorological patterns can vary considerably over short distances due to spatial heterogeneity of the terrain (Kalashnikov et al., 2020). *Second*, with few exceptions (e.g., Allen & Pickering, 2002), most previous studies have parameterized total lightning flash rate (including intra‐cloud and CG) and occasionally estimated CG lightning in future projections using empirically derived ratios of CG to total lightning (Krause et al., 2014; Pérez‐Invernón et al., 2023; Price & Rind, 1994). Here we train CNNs to explicitly predict CG lightning across the WUS as only this type of lightning poses the risk of wildfire ignition.

## 2 Data and Methods

### 2.1 Data Sets

Daily gridded CG lightning flash totals (0.1° × 0.1°; 1995–2022) are obtained from the National Lightning Detection Network (NLDN). The NLDN is a ground‐based lightning sensor network with a reported detection efficiency of 95% (Nag, 2014) and CG stroke classification accuracy of 92% (Vagasky et al., 2024). Meteorological data at 1‐ and 3‐hourly resolution are obtained or derived from the National Aeronautics and Space Administration's (NASA) Modern‐Era Retrospective Analysis for Research and Applications, Version 2 (MERRA‐2; Gelaro et al., 2017) that has a spatial resolution of 0.5° latitude × 0.625° longitude. All data sets are upscaled, as described below, to 1° × 1° spatial resolution to create a larger sample size of CG lightning days in each grid cell to train the CNNs, while still capturing sub‐regional variations in CG lightning activity. This resolution is additionally comparable to GCMs that are mostly output on 1° × 1° or coarser grids. A day in each 1° × 1° grid cell is characterized as a CG lightning day if any of the constituent 0.1° × 0.1° NLDN grid cells experience CG lightning. Non‐lightning days are days without any CG lightning in any constituent grid cell. MERRA‐2 data are bilinearly interpolated and averaged to daily values to match temporally and spatially with the CG lightning data at 1° resolution.

### 2.2 Study Domain

**Figure 1:**
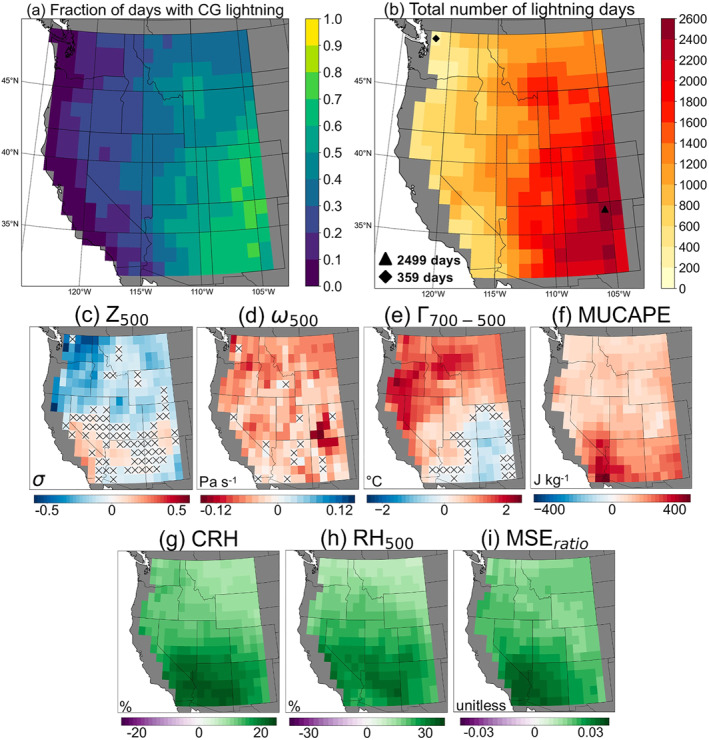
(a) Fraction and (b) total number of days with at least one CG lightning flash in 1° × 1° grid cells of the WUS during June–September (1995–2022). Markers and inset text in panel (b) denote the grid cells with the most and least cumulative CG lightning days between 1995 and 2022. (c–i) Differences in local meteorological variables on CG lightning days relative to days without CG lightning. Hatching indicates that differences are insignificant (*p* ≥ 0.05) between CG lightning and non‐lightning days according to a two‐tailed *t*‐test. Areas along the Pacific coast shaded in gray in panels (b–i) are excluded from further analysis due to low sample size of CG lightning days (<10% of all days).

We construct CNNs to predict the daily occurrence of CG lightning in each 1° × 1° grid cell over the conterminous WUS (Figure 1; 32°–49°N and Pacific coast to 104°W) over June–September (hereafter, “warm season”). We exclude grid cells that experience CG lightning on less than 10% of warm‐season days (i.e., CG lightning fraction of <0.1), corresponding to Pacific coastal areas where lightning‐caused wildfire ignitions are rare (Figures 1a and 1b; Brey et al., 2018; Kalashnikov et al., 2023). The total number of CG lightning days and corresponding CG lightning fraction are highest over the interior Southwest, as these areas are directly influenced by convection associated with the North American monsoon (Adams & Comrie, 1997; Barlow et al., 1998). The most CG lightning days over the study period occurred at a grid cell in northern New Mexico (*n* = 2499), representing ∼73.2% of all warm‐season days (Figure 1b).

### 2.3 Meteorological Variables

We utilize a set of seven dynamic and thermodynamic variables that influence CG lightning occurrence, with variable selection informed by literature (Table S1 in Supporting Information S1; Figures 1c–1i). These variables broadly describe moisture availability, atmospheric instability, and the large‐scale atmospheric circulation. Atmospheric circulation is captured through deseasonalized, standardized anomalies of 500 hPa geopotential height (Z_500_), computed from 7‐day windows centered on each calendar date in the 28‐year record. To characterize atmospheric moisture, we use column‐integrated relative humidity (CRH), defined as the ratio of vertically integrated atmospheric water vapor to its saturation counterpart (Mo et al., 2021; Wolding et al., 2020). CRH is analogous to total column water vapor but is a more direct proxy for liquid and ice content, as the amount of water vapor that can condense depends on the degree of saturation (Etten‐Bohm et al., 2021; Mo et al., 2021). We also use 500 hPa relative humidity (RH_500_) to capture “high‐based” convection commonly associated with WUS thunderstorms, particularly outside the core monsoon region of the interior Southwest (Kalashnikov, Abatzoglou, et al., 2022; Krumm, 1954; Nauslar et al., 2013; Rorig et al., 2007). In these situations, moisture advection and cloud bases can be above 700 hPa and convection does not depend on the moisture profile below that level, thus limiting the usefulness of total‐column quantities. Synoptic‐scale ascent is quantified through omega, or vertical velocity, at 500 hPa (ω_500_) and potential updraft strength through the vertical temperature difference, or lapse rate, between the 700 and 500 hPa pressure levels (Γ_700‐500_). We also evaluate a larger set of variables as potential predictors, including omega and relative humidity at 700 hPa. Differences in these variables at 500 hPa on CG lightning relative to non‐lightning days were more prominent than at 700 hPa across most of the domain (not shown).

We also use two derived variables that combine temperature and moisture into integrated metrics of instability that are indicators of environments favorable for convection: “most unstable” convective available potential energy (MUCAPE) and the ratio of surface moist static energy to the free‐troposphere saturation moist static energy (MSE_*ratio*_). Our rationale for using MUCAPE over surface‐based CAPE is that high‐based convection can occur independently of surface‐based instability (or lack thereof) in certain environments due to mid‐tropospheric moisture and instability overlying a stable boundary layer, and previous studies have found CAPE to be relatively unimportant for lightning prediction over the WUS (Burrows et al., 2005; Liu et al., 2022). Rather than integrating from the surface, MUCAPE is integrated vertically from the most unstable parcel in the lowest 300 hPa to the equilibrium level, and is therefore less sensitive to situations when convection initiates above the surface layer (Luong et al., 2017; Rochette et al., 1999). We compute MUCAPE using the *MetPy* Python package (version 1.4.0; May et al., 2023). MSE is a thermodynamic quantity that combines temperature and moisture information and is conserved during adiabatic motions. The MSE_*ratio*_ can be used to diagnose convection, which occurs when the surface air parcel MSE is equal to the free‐troposphere saturation MSE (e.g., MSE_*ratio*_ = 1). Our use of MSE_*ratio*_ is informed by its recent application in studies that sought to define a theoretical upper bound for near‐surface temperatures by considering the convective (in)stability of the boundary layer (Noyelle et al., 2023; Zhang & Boos, 2023). More details on the derivation of CRH and MSE_*ratio*_ can be found in the Supporting Information S1.

Our overarching goal with the selection of these seven variables is to ensure that (a) they are useful at differentiating CG lightning from non‐lightning conditions across the WUS, and (b) they are “climate‐invariant” (Beucler et al., 2024; Molina et al., 2021), meaning their relationship with CG lightning should be generalizable to a future, warmer climate in GCM projections. This entails using moisture quantities that are relative, rather than absolute, as the latter can be influenced by underlying shifts due to warming. For example, using measures of relative humidity (e.g., CRH and RH_500_) is preferable to water vapor content and specific humidity. This is because future saturation specific humidity will increase with temperature through the Clausius‐Clapeyron relation, changing the saturation profile for a given amount of atmospheric water vapor and likely affecting the point at which convection is triggered (Beucler et al., 2024). Meanwhile, constant relative humidity is expected with warming (Douville et al., 2022). As proxies for upward vertical motion, ω_500_, Γ_700‐500_, MUCAPE, and MSE_*ratio*_ should remain physically consistent over time. Finally, use of Z_500_ standardized anomalies preserves gradients in the anomaly fields irrespective of background tropospheric expansion, as future values would be calculated from a baseline period from that climate.

Figures 1c–1i shows the differences in the local values of meteorological variables between CG lightning and non‐lightning days at each grid cell (see Figure S1 in Supporting Information S1 for composite values on CG lightning days). We use a two‐tailed *t*‐test (*p* < 0.05) to test the significance of difference in meteorological variables on days with CG lightning versus non‐lightning days. MUCAPE, CRH, RH_500_, and MSE_*ratio*_ are uniformly and significantly higher (*p* < 0.05) across the WUS on CG lightning days compared to non‐lightning days, indicating more moisture and energy available for convection. However, magnitudes of these differences vary and are larger in areas to the south that are under the direct influence of the North American monsoon. ω_500_ is generally more negative on CG lightning days compared to non‐lightning days indicating stronger upward vertical motion in the mid‐troposphere (Figure 1d). In contrast, Z_500_ and Γ_700‐500_ show anomalies of the opposite sign between northern and portions of southern areas (Figures 1c and 1e). Lightning activity in areas on the North American monsoon periphery, including the interior Northwest and northern Rocky Mountains, is frequently associated with mid‐latitude disturbances which induce significantly lower Z_500_ and steeper Γ_700‐500_ through cold air advection aloft (Kalashnikov et al., 2020; Werth & Ochoa, 1993). The lack of steepened Γ_700‐500_ on CG lightning days in parts of the interior Southwest is likely due to a seasonally unstable atmosphere that does not require large deviations from average conditions to initiate convection (Adams & Souza, 2009; Kalashnikov et al., 2020).

### 2.4 CNN Architecture, Training, and Tuning

**Figure 2:**
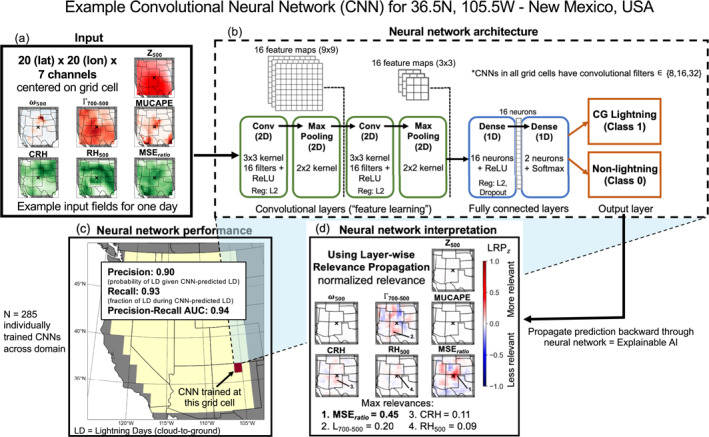
Example convolutional neural network (CNN) trained at a grid cell in New Mexico (corresponding to the location with the largest number of CG lightning days in Figure 1b and denoted with black “x” in panels (a, d)) showing (a) input variables for one day interpolated to 2000 × 2000 km equal area grids centered on that grid cell, (b) schematic diagram of model architecture, and (c) model performance. (d) Composite relevance maps of each input variable for CG lightning predictions (Class 1), calculated using LRP_*z*_. Red shading indicates positive contributions (relevance) to successful CG lightning predictions, with higher values indicating increased relevance. Inset numbers and marker lines in panel (d) indicate locations of maximum relevance for MSE_*ratio*_, Γ_700‐500_, CRH, and RH_500_, with corresponding values shown below (Z_500_, ω_500_, and MUCAPE not shown due to low relevance).

We train individual CNNs at each 1° × 1° grid cell (*n* = 285) to predict CG lightning occurrence using the seven meteorological variables as inputs (Figure 2). In this work, our CNNs are binary classifiers that automatically learn spatial patterns from the input data and output a probability of CG lightning (Class 1) or non‐lightning (Class 0). Daily fields of each meteorological variable are interpolated to equal‐area 20 × 20 grids spanning 2000 km on each side (100 km × 100 km resolution), and centered on the grid cell for which the CNN is trained. This was done by sampling the underlying meteorological fields at points spaced every 100 km in the *x* and *y* directions from the center of each 1° × 1° grid cell (i.e., each local origin). This procedure necessitates a larger underlying field from which the input variables are resampled that extend beyond the WUS boundaries: for example, the input field around the grid cell in New Mexico extends south into Mexico and eastward to Iowa (Figure 2a). The 2000 km distance is chosen to roughly approximate a Rossby half‐wavelength (Stoll et al., 2023), thereby capturing large‐scale circulation features pertinent for CG lightning. Although this distance could create unnecessarily large fields for thermodynamic variables, the CNNs will learn to select the most relevant spatial features within these domains during training (Baño‐Medina et al., 2021). For each day, the input to each local CNN thus consists of a three‐dimensional matrix (20 × 20 × 7; lat *x* lon *x* input variables) (Figure 2a). To increase stability and robustness during training, all input variables are rescaled by their maximum value across the full domain and time period. Training labels provided to the CNNs are the presence/absence of CG lightning from the NLDN in that 1° × 1° grid cell.

We start with the same architecture for each CNN, but perform hyperparameter tuning separately for each. See Table S2 in Supporting Information S1 for a complete listing of tuned hyperparameters and the range of values over which optimization is conducted. By adopting a comprehensive hyperparameter tuning strategy for each CNN, we seek to refine the approach of Cheng et al. (2024), who used a single CNN architecture for all grid cells with a limited subset of tuned hyperparameters (a full description of the hyperparameter tuning process can be found in the Supporting Information S1). The CNNs each have two convolutional layers that process input data utilizing 3 × 3 filters, each followed by a “max pooling” layer utilizing 2 × 2 filters. The output of the convolutional and max pooling layers is then passed to a dense layer with 16 neurons. An example of the CNN architecture for one location is shown in Figure 2b. We use He uniform initialization (He et al., 2015), and the convolutional and first dense layer use the rectified linear unit (ReLU) activation function. As a final step, inputs are vectorized and passed to a classification layer with two neurons and a softmax activation function. Output from this layer consists of continuous probabilities of each classification (CG lightning or non‐lightning) on that day scaled to a range of [0, 1]. We define predicted CG lightning days as all days with a model‐predicted CG lightning probability >0.5.

The CNNs are trained using 70% of the observed data (2391 days), with equal portions of the remaining 30% of the data withheld for validation and testing (Labe & Barnes, 2022). The validation data set is used for hyperparameter tuning and early stopping during training, while the test data set is withheld to assess the models' ability to generalize to unseen data. Due to class imbalance in many grid cells (Figure 1a), we use a stratified random split to ensure that training, validation, and testing data sets have the same ratio of CG lightning to non‐lightning days. We train the CNNs using categorical cross‐entropy as the loss function and the Adam gradient‐descent optimizer (Kingma & Ba, 2014). Results obtained by using the RMSprop optimizer are similar (not shown). We use early stopping to end model training when validation loss begins to increase (patience = 10 epochs), which can help limit overfitting (Goodfellow et al., 2016). The CNNs are built and trained using *TensorFlow 2.11.1* in Python (Abadi et al., 2015).

We use three metrics for evaluating the performance of the CNNs. *Precision* quantifies the fraction of CNN‐predicted CG lightning days that actually observed CG lightning. We also quantify *recall*, also known as the probability of detection, which quantifies the fraction of observed CG lightning days that were correctly detected by the CNN, and the *precision‐recall area under the curve (AUC)*, which synthesizes precision and recall into a single model performance metric (Figure 2c; an AUC >0.5 indicates predictive skill that is better than random guessing). The precision‐recall AUC is preferable to receiver operating characteristic AUC in this application due to the class imbalance in many grid cells (Davis & Goadrich, 2006). For each CNN, hyperparameters are tuned to maximize precision on the validation data set. This choice of optimization metric is subjective and depends on the scientific problem (Davenport & Diffenbaugh, 2021). In our application, we seek to minimize false positives as this reduces overprediction of CG lightning.

### 2.5 CNN Explainability

Although the primary goal of this study is to predict CG lightning, we are also interested in understanding how the CNNs utilize the information contained in the predictor variables as this can yield physical insights. We use LRP, a post‐hoc XAI technique, to interpret the CNN classifications following previous studies (Davenport & Diffenbaugh, 2021; Labe & Barnes, 2021, 2022; Mamalakis, Barnes, & Ebert‐Uphoff, 2022; Mamalakis, Ebert‐Uphoff, & Barnes, 2022; Toms et al., 2020). In this procedure, the CNNs are first trained, and the weights and biases are frozen. Then, daily predictions are propagated backward through the network while keeping track of the relevance of information passed between neurons in different layers (Montavon et al., 2019). For each predictor variable, a heatmap of relevance scores is produced wherein locations with higher values indicate that their information was more relevant to the prediction. LRP is computed using the *iNNvestigate* Python package (version 2.0.2; Alber et al., 2019).

Although relevance maps are generated for all days including CG lightning and non‐lightning predictions, we focus on visualizing relevance for CG lightning predictions only. We use the LRP_*z*_ variant (utilizing the *z*‐rule for propagation; Bach et al., 2015) that allows us to track both positive and negative relevance (Labe & Barnes, 2022; Mamalakis, Ebert‐Uphoff, & Barnes, 2022). We also compute relevance using a composite LRP variant (LRP_*comp*_) following recommendations in Kohlbrenner et al. (2020). We implement LRP_*comp*_ using “LRPSequentialPresetA” from the *iNNvestigate* package, which consists of LRP_*alpha‐beta*_ (α = 1, β = 0) in the convolutional layers and LRP_*epsilon*_ (ε = 0.1) in the fully connected layers. Note that LRP_*z*_ is equivalent to the Input*Gradient XAI technique (Shrikumar et al., 2016) for neural networks that use ReLU activation (Mamalakis, Barnes, & Ebert‐Uphoff, 2022). Ultimately, the choice of XAI technique is subjective and no single method has consistently proven optimal (Bommer et al., 2023; Mamalakis, Barnes, & Ebert‐Uphoff, 2022; Molina et al., 2023; Sixt et al., 2019). Relevance maps are summed by grid cell for all CG lightning days in the period of record, and the summed values are further normalized across the predictor space to a range of [−1, 1] for comparison of relative relevance to successful CG lightning prediction. Since LRP_*z*_ output is inherently noisy and can be difficult to interpret (Kohlbrenner et al., 2020; Montavon et al., 2019), the final LRP_*z*_ maps are smoothed with a Gaussian filter (sigma = 0.8) to reduce noise. Example LRP_*z*_ maps for the local CNN trained to predict CG lightning at 36.5°N, 105.5°W are shown in Figure 2d. To enable comparison of LRP values across all 285 CNNs, we extract the maximum LRP_*z*_ value for each variable (Figure 2d). For example, for this grid cell the maximum LRP_*z*_ value is associated with MSE_*ratio*_ (0.45) and is located at a pixel adjacent, and slightly northeast, of the lightning location (Figure 2d).

### 2.6 Logistic Regression

We compare the performance of CNNs in predicting CG lightning occurrence with logistic regression models (Bliss, 1935; McCullagh & Nelder, 1989) constructed at each 1° × 1° grid cell in the WUS to evaluate whether the CNNs provide an advantage over a traditional classification method. Logistic regression is a type of generalized linear model that, similar to CNNs, outputs a continuous probability of CG lightning occurrence for each day. We utilize the same seven meteorological variables as predictors. However, a key difference from CNNs is that inputs to the logistic regression consist only of the local value of each predictor from the grid cell for which the prediction is made, rather than the surrounding spatial fields. Logistic regression has been widely used for the prediction of climate phenomena, both as a primary tool and as a baseline to compare with other machine learning approaches (Bates et al., 2018; Chattopadhyay et al., 2020; Etten‐Bohm et al., 2021; Jergensen et al., 2020; Kamangir et al., 2020; Labe et al., 2023; Mayer & Barnes, 2021). Similar to the CNNs, we apply a hyperparameter tuning approach that seeks to maximize precision. We tune the L2 regularization factor and the magnitude of the class weights, and evaluate results using 4‐fold cross validation. Logistic regression models are built and trained using the *scikit‐learn* Python package (version 1.2.2; Pedregosa et al., 2011).

## 3 Results and Discussion

### 3.1 CNN Performance and Interpretation

**Figure 3:**
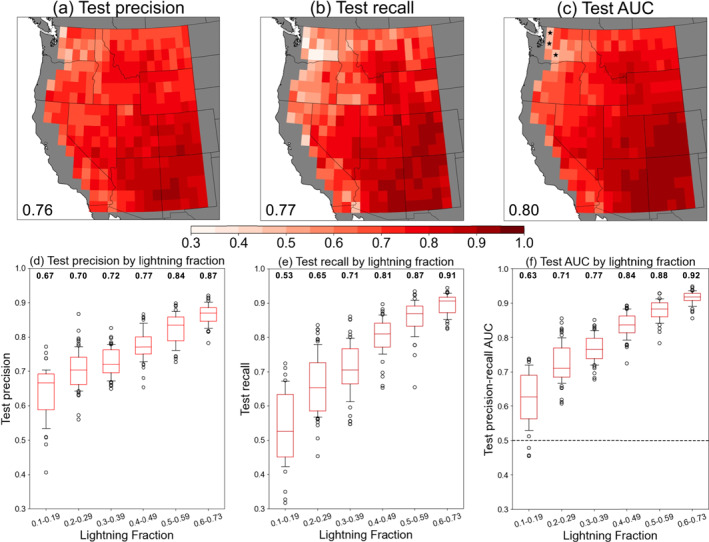
Performance metrics on test data sets for all convolutional neural networks individually trained in 1° × 1° grid cells (*n* = 285) quantified through (a) precision, (b) recall, and (c) precision‐recall AUC. Inset text shows domain‐median values. (d–f) Performance metrics binned by CG lightning fraction. Values above boxplots are the medians for each fraction bin. Black markers in panel (c) denote grid cells where AUC <0.5, indicating no predictive skill.

The three performance metrics for all 285 local, individually trained CNNs (one at each 1° × 1° grid cell) are shown in Figure 3, and are obtained from the 15% of data that were withheld for testing at each grid cell. The median precision across the WUS is 0.76, recall is 0.77, and the AUC is 0.8 (Figures 3a–3c). Across most of the WUS, AUC is substantially higher than 0.5, indicating that local CNNs are skillful classifiers of CG lightning versus non‐lightning days. Further, the locally trained CNNs outperform a single CNN that was trained on all grid cells (AUC = 0.69; Figure S2 in Supporting Information S1). Spatial variation in CNN performance is apparent and exhibits a nearly monotonic increase with increasing CG lightning fraction (Figures 3d–3f). The best‐performing models are in grid cells where CG lightning occurs on ≥60% of all warm‐season days (Figure 1a) with a median precision/recall/AUC of 0.87/0.91/0.92, respectively (Figures 3d–3f). This region encompasses Colorado and New Mexico and is under the direct influence of the North American monsoon during the mid‐late summer period, with frequent convective activity and the largest sample size of CG lightning days for CNN training.

The lowest performance is found in grid cells where CG lightning is rare, occurring on 10%–19% of warm‐season days (Figure 1a) and located in portions of interior Washington, Oregon, and California. In these grid cells, the median precision/recall/AUC is 0.67/0.53/0.63, respectively (Figures 3d–3f). The lower classification performance in areas of infrequent lightning activity is similar to results obtained by Bates et al. (2018) for Australia, who tested six statistical classifiers for lightning prediction. The underperformance is therefore unlikely to be CNN‐dependent, but could arise from an insufficient sample size of lightning days for statistical learning regardless of the method. Further, it is also possible that the chosen set of meteorological predictors may not fully describe lightning conditions here. This region lies at the periphery of the monsoonal circulation and is prone to high‐based “dry” thunderstorms, which can produce lightning from narrow layers of elevated moisture and instability that may not be captured by our analysis (Abatzoglou et al., 2016; Kalashnikov, Abatzoglou, et al., 2022; Nauslar et al., 2013).

**Figure 4:**
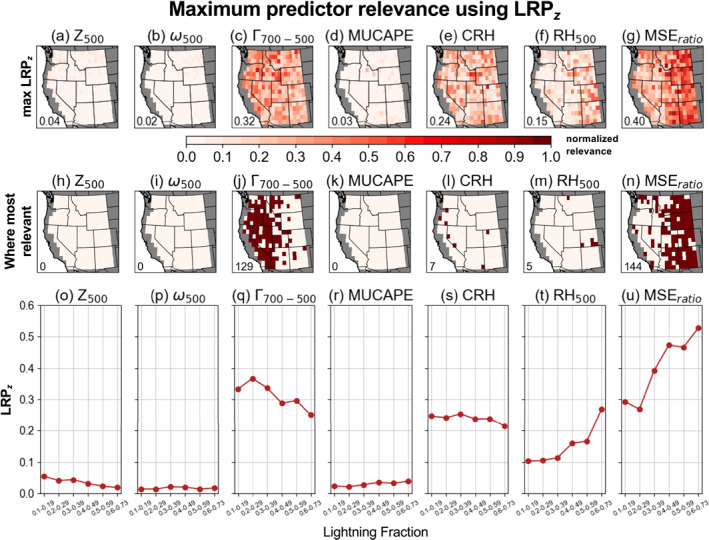
(a–g) Maximum relevance of each predictor for successful CG lightning‐day predictions for each convolutional neural network (CNN) computed from spatial fields of LRP_*z*_. Higher values indicate increased relevance. Inset text shows mean domain‐wide values of maximum relevance normalized to a scale of 0–1. (h–n) For each predictor, location of grid cells (maroon shading) where this predictor has highest relevance for successful CG lightning‐day predictions in the corresponding CNN compared to the other variables. Inset text shows total number of CNNs where that predictor was most relevant. (o–u) Mean values of maximum LRP_*z*_ binned by lightning fraction.

We assess the relevance of individual predictor variables to successful CG lightning‐day predictions for each CNN (Figure 4). For each variable and grid cell, the relevance maps show the maximum LRP_*z*_ (smoothed, see Section 2) associated with CG lightning‐day predictions for the CNN trained at that location. MSE_*ratio*_ has the highest domain‐averaged maximum‐LRP_*z*_ of 0.4, and is particularly high in the eastern half of the domain (Figures 4g and 4u). Γ_700‐500_ has the second‐highest maximum‐LRP_*z*_ of 0.32, with higher values in central and western portions of the domain (Figures 4c and 4q). MSE_*ratio*_ is the most relevant predictor variable for 144 of the CNNs (∼51%; Figure 4n), mainly in the eastern half of the domain, whereas Γ_700‐500_ is the most relevant for an additional 129 CNNs (∼45%; Figure 4j) mainly in the western half of the domain. Together, these variables represent the most relevant CG lightning predictors for 273 of the 285 CNNs (∼96%). The increased relevance of Γ_700‐500_ in areas further west is consistent with steepened mid‐tropospheric lapse rates associated with CG lightning days outside the North American monsoon core (Figure 1e), which promote convection by increasing mid‐level instability (Kalashnikov, Abatzoglou, et al., 2022; Kalashnikov et al., 2020). The increased relevance of MSE_*ratio*_ in eastern areas is likely related to their location under the direct influence of the North American monsoon. MSE_*ratio*_ is a relatively simple, surface‐based proxy for convection and may sufficiently describe conditions favorable for CG lightning in this region where moisture, sensible heating, and orographic lifting for parcel ascent are frequently present during monsoon season (Adams & Souza, 2009).

**Figure 5:**
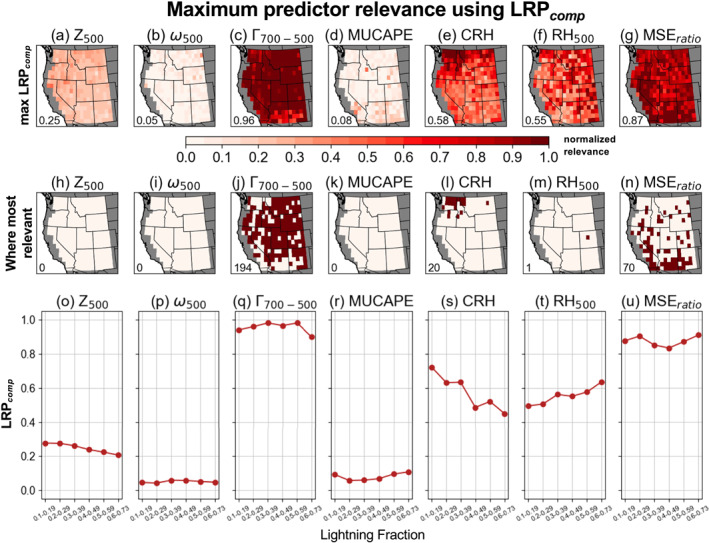
As in Figure 4, but using LRP_*comp*_ for predictor relevance.

Results obtained from LRP_*comp*_ are similar with MSE_*ratio*_ and Γ_700‐500_ representing the most relevant predictors for ∼93% of CNNs (Figure 5). However, Γ_700‐500_ is the most relevant predictor using LRP_*comp*_ when averaged across the WUS (Figure 5c; note that absolute relevance values of LRP_*z*_ and LRP_*comp*_ are not directly comparable due to different propagation rules). Another notable difference when using LRP_*comp*_ compared to LRP_*z*_ is that CRH emerges as the most relevant predictor for 20 CNNs (∼7%; Figure 5l), largely concentrated in the interior Northwest. The other predictor variables—Z_500_, ω_500_, and MUCAPE—show much lower relevance for CG lightning predictions across the WUS when using both LRP_*z*_ and LRP_*comp*_ (Figures 4a, 4b, 4d and 5a, 5b, 5d). These findings may be counterintuitive, particularly for MUCAPE, which is widely used to quantify convective environments both in research and in operational forecasting. We note that it is important to distinguish between predictor relevance from the perspective of CNNs attempting to predict CG lightning and actual physical importance for convection and lightning production. Since our goal is to predict the daily occurrence of CG lightning (≥1 CG flash), it is likely that the CNNs prioritized the information contained in relatively simple convective proxies like MSE_*ratio*_ and Γ_700‐500_ during the training process. If predicting lightning flash density, MUCAPE would likely emerge as a more important predictor, as the energy available for convection would be expected to correlate with increased lightning production when convection is triggered (e.g., Romps et al., 2014). We also construct a reduced version of these CNNs consisting of three predictors (MSE_*ratio*_, Γ_700‐500_, and RH_500_) that could be applied to GCMs to reduce computational burden. Performance is comparable overall (domain‐median AUC = 0.79) and in the Southwest, but somewhat degraded in parts of the interior Northwest (Figure S3 in Supporting Information S1).

### 3.2 Comparison With Logistic Regression

**Figure 6:**
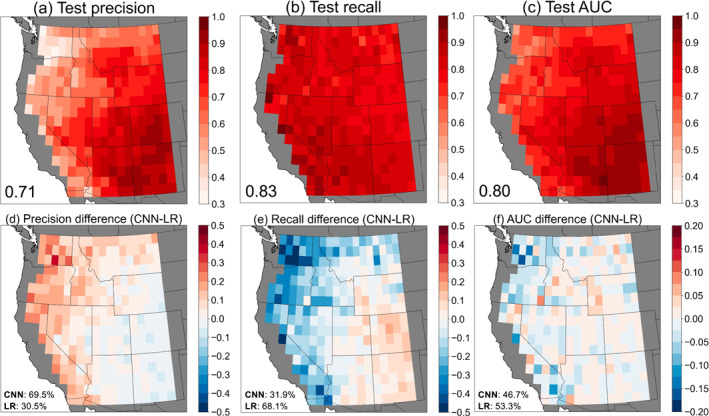
(a–c) Performance metrics as in Figure 3, but for logistic regression models. (d–f) Differences between convolutional neural networks and logistic regression across the domain. Inset text shows the percent of grid cells that had better performance for each model and metric.

We also compare results obtained from CNNs with those obtained from logistic regression models similarly trained at each 1° × 1° grid cell and optimized for precision. Overall, logistic regression performs comparably when aggregated across the WUS (median precision/recall/AUC of 0.71/0.83/0.8, respectively; Figures 6a–6c). However, CNNs demonstrate improvement in precision across most of the WUS (in ∼69.5% of grid cells; Figure 6d). Higher precision for CNNs is widespread and pronounced across the western and northern periphery, where some grid cells exhibit a >20% increase compared to logistic regression (Figure 6d). Our CNNs therefore help mitigate false positives in lightning classification by ensuring that a larger fraction of predicted CG lightning days observe CG lightning. We hypothesize that our spatial CNNs outperform local logistic regression in regions on the periphery of the North American monsoon as they additionally capture the spatial patterns of meteorological predictors important in those areas. However, some of these differences may result from the different levels of complexity in the hyperparameter tuning applied to CNNs versus logistic regression when optimizing for precision, as CNNs have more tunable parameters. Conversely, CNNs lead to generally lower recall (i.e., more false negatives or CG lightning occurring on predicted “non‐lightning” days) compared to logistic regression (∼68% of grid cells have higher recall using logistic regression; Figure 6e). AUC values are generally similar, with ∼47% of grid cells observing higher values using CNNs and ∼53% using logistic regression (Figure 6f). These results indicate that when considering overall model performance, CNNs do not offer a systematic advantage over logistic regression across the domain, and the best type of model to parameterize CG lightning may vary by location.

### 3.3 CG Lightning Parameterization

**Figure 7:**
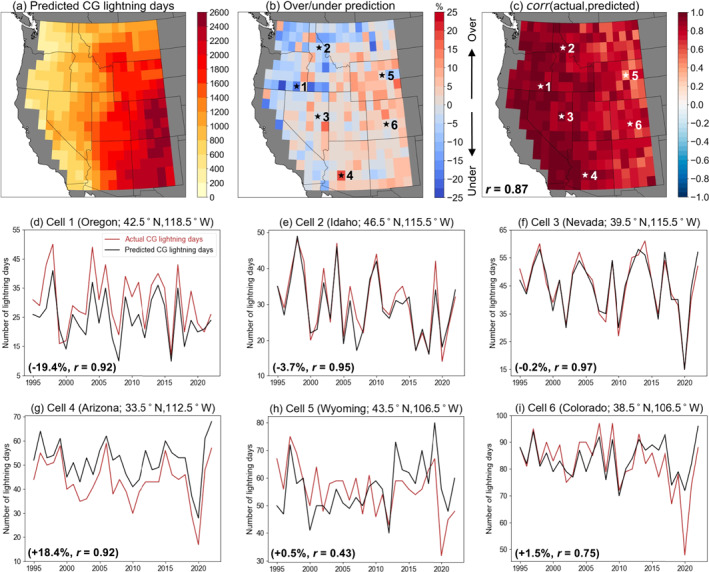
(a) Total number of CG lightning days predicted by locally trained convolutional neural networks between June–September (1995–2022), and (b) corresponding prediction error compared to observations. (c) Interannual correlation between actual and predicted CG lightning days, with inset text showing domain median. (d–i) Time series of actual and predicted CG lightning days for six example grid cells, with locations indicated in panels (b, c). Text in the bottom left in panels (d–i) refers to the corresponding prediction errors and Pearson's correlation coefficients.

We apply the trained CNNs to meteorological fields over the full 1995–2022 period and generate predictions of CG lightning days (Figure 7). Figure 7a shows the total number of predicted CG lightning days over the study period and their spatial patterns. The geographical distribution of CNN‐predicted CG lightning days is very similar to observations in Figure 1b, as the CNNs realistically capture the northwest‐to‐southeast gradient of increasing CG lightning activity across the WUS (Figure 7a). CNNs generally underpredict CG lightning days compared to NLDN observations in the Pacific Northwest, western Great Basin, and northern Rockies where lightning is relatively rare (Figure 7b; Figure S4a in Supporting Information S1). However, this underprediction could stem from our decision to optimize the CNNs for precision, which limits false positives but can lead to underprediction. Meanwhile, CNNs overpredict CG lightning days in the rest of the interior WUS where lightning occurs more frequently.

Across all CNNs, the differences between the total number of predicted CG lightning days and the number of observed CG lightning days are within 25% (Figure 7b). The CNNs additionally capture the seasonal cycle of CG lightning activity, with a pronounced peak in predicted CG lightning days over the interior Southwest in July–August similar to observations (not shown). Our results demonstrate that CNNs are able to skillfully reproduce the long‐term climatology of CG lightning days across the WUS over the historical period. Further, the locally trained CNNs developed in this study appear to be more skillful at predicting CG lightning over the WUS compared to recent large‐scale lightning parameterization efforts over the tropics and subtropics. For example, Etten‐Bohm et al. (2021) used a global logistic regression model and reported widespread underprediction of lightning days. Similarly, using regionally trained random forest models to predict individual thunderstorms, Liu et al. (2022) noted large biases between predictions and observations locally exceeding 100%.

Correlation coefficients between the actual and predicted number of CG lightning days during our study season each year (i.e., interannual correlation) are shown in Figure 7c. The CNNs realistically capture year‐to‐year variations in the number of CG lightning days across the WUS when compared to observations, as all correlation coefficients are between 0.43 and 0.99 (median *r* = 0.87). Nearly 40% of CNNs (113 of 285) have interannual correlation >0.9, largely concentrated in the western half of the domain (Figure 7c; Figure S4b in Supporting Information S1). Generally lower correlation is found in the eastern half of the domain, where the median correlation reduces to 0.73 in grid cells with ≥60% CG lightning fraction (Figure S4b in Supporting Information S1). These results represent a reversal of the spatial patterns in Figure 3, wherein higher model performance (based on classification metrics) is found in southeastern areas with lower performance further west. One possible explanation is that areas further west are reliant on transient episodes of favorable moisture and instability conditions to generate lightning activity, as they lie outside the monsoon core (Abatzoglou & Brown, 2009; Kalashnikov et al., 2020; Nauslar et al., 2013; van Wagtendonk & Cayan, 2008). Consequently, the frequency of these episodes is subject to higher interannual variability that may be easier for the CNNs to discern over seasonal timescales, compared to locations further east, resulting in higher interannual correlation with observations despite lower classification performance at the daily timescale (Figure 3). Our results compare favorably with Cheng et al. (2024), who used a single neural network to predict lightning flash density in the WUS and reported a 0.57 correlation with observations. The stronger correlation observed in this study demonstrates the effectiveness of locally trained CNNs in accurately predicting lightning days on a seasonal basis.

We compare the actual and CNN‐predicted CG lightning days over 1995–2022 at six example grid cells (Figures 7d–7i), with the aim of studying cases of success and failure (Ebert‐Uphoff & Hilburn, 2020). The CNN at Cell 1 (southeastern Oregon; Figure 7d) represents an example of high interannual correlation (*r* = 0.92) but with consistent underprediction (−19.4% over the period of record). Conversely, the CNN at Cell 4 (Arizona; Figure 7g) is an example of high interannual correlation (*r* = 0.92) but with consistent overprediction (+18.4%, largest in the domain). Cell 2 (northern Idaho; Figure 7e) and Cell 3 (Nevada; Figure 7f) are examples of both high interannual correlation (*r* = 0.95 and 0.97, respectively) and low prediction error (−3.7% and −0.2%), and highlight the ability of the locally trained CNNs to accurately reproduce the climatology of CG lightning days. On the other hand, the CNN at Cell 5 (Wyoming; Figure 7h) exhibits systematic underprediction until 2011 and overprediction in following years despite capturing most of the peaks in CG lightning activity (Figure 7h). The CNNs at this grid cell and Cell 6 (Colorado, Figure 7i) are notable for their inability to predict the magnitude of anomalously low CG lightning activity in 2020. The 2020 monsoon season was historically dry in the interior Southwest with limited convective activity (Hoell et al., 2022; Ren et al., 2022), and resulted in the lowest number of CG lightning days at five of the six example grid cells (all except Cell 1; Figures 7e–7i). However, the lack of CG lightning days was better predicted by the CNNs further west, particularly in Cells 2 and 3 in Idaho and Nevada, respectively (Figures 7e and 7f).

To understand the systematic overprediction by the CNN for Cell 4 in Arizona, we compare composite values of the predictor variables for this CNN on false positive days, when CG lightning was predicted but did not occur, with values on true positive days when CG lightning was correctly predicted (Figure S5 in Supporting Information S1). On false positive days, quantities of moisture and convective energy are suppressed compared to true positive days, as MUCAPE, CRH, RH_500_, and MSE_*ratio*_ are significantly lower (*p* < 0.05) over the grid cell and surrounding region (Figures S5d–S5g in Supporting Information S1). The absolute values of these variables are still generally high on false positive days (not shown), likely signaling to the CNN that at least marginally favorable conditions for convection exist and making it difficult to distinguish from true positive days. Values of Z_500_, Γ_700‐500_, and ω_500_ are generally higher on false positive days but these differences are insignificant near the lightning location in all three cases (Figures S5a–S5c in Supporting Information S1).

### 3.4 Prediction Confidence

**Figure 8:**
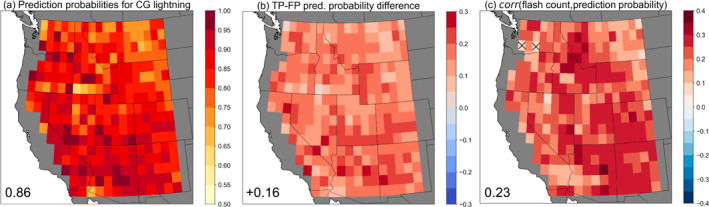
(a) Median prediction probabilities (“confidence”) for all days on which CG lightning was predicted by locally trained convolutional neural networks in each grid cell. (b) Differences in CG lightning‐day prediction confidence between true positive days (TP) and false positive days (FP). (c) Correlation coefficients between CG lightning‐day prediction confidence and the total number of observed CG lightning flashes on that day. Hatching in panel (c) indicates that correlation is insignificant (*p* ≥ 0.05). Inset text shows domain‐median values.

As CNNs output a continuous probability of CG lightning for each day, we examine this prediction probability (“confidence”) on all predicted CG lightning days (i.e., days with a predicted CG lightning probability >0.5). In performing this analysis, we ask the question of whether CG lightning days with high prediction confidence (closer to 1) exhibit meaningfully different characteristics compared to days with lower prediction confidence (closer to 0.5). The CNNs generally predict CG lightning with high confidence across the WUS (median = 0.86; Figure 8a). Prediction confidence tends to be higher in the western parts of the domain and lower over Montana and Wyoming, where the geography shifts to the Great Plains with different underlying topography, land surface, and climatology compared to the rest of the WUS. Days when CG lightning was correctly predicted have a systematically higher prediction confidence compared to days when CG lightning was falsely predicted across all CNNs (Figure 8b). This finding confirms that CNN predictions are more likely to be physically correct with higher prediction confidence, in agreement with Mayer and Barnes (2021).

Further, all CNNs exhibit positive correlation between prediction confidence and the total *quantity* of CG lightning flashes on that day. This correlation, although weak (domain median *r* = 0.23), is significant at all but two CNNs in the WUS (Figure 8c). We also analyze the top‐10% most confident CG lightning‐day predictions for each CNN, defined as the predicted CG lightning days with >90th percentile prediction probability (Mayer & Barnes, 2021). CG lightning flash counts on days with the top‐10% most confident CG lightning predictions are typically higher by a factor of 2.47 (domain median) compared to days with less confident CG lightning predictions (<90th percentile prediction probability; Figure S6 in Supporting Information S1). These results carry important implications for applying our CNN‐based CG lightning parameterizations to GCMs, as an increase in CG lightning flash quantity can be expected for more confident predictions in future climates, leading to refined projections of wildfire ignition risk when weather and fuel conditions align.

## 4 Summary and Conclusions

In this study, we develop locally trained CNNs at each 1° × 1° grid cell in the WUS to predict the daily occurrence of CG lightning over June–September 1995–2022. CG lightning is an important ignition mechanism for wildfires across this region, causing 40% of ignitions and 69% of the total burned area in the interior WUS in recent decades (Abatzoglou et al., 2016). Two‐dimensional fields of seven dynamic and thermodynamic meteorological variables are used as predictors in the CNNs: Z_500_, ω_500_, Γ_700‐500_, MUCAPE, CRH, RH_500_, and MSE_*ratio*_. All seven variables show significant differences between CG lightning and non‐lightning days across most of the domain, with values of moisture and instability significantly larger on CG lightning days (Figure 1). The locally trained CNNs are skillful at predicting CG lightning occurrence across the WUS (median AUC = 0.8) and perform best in parts of the interior Southwest where summertime lightning is most common (AUC >0.9) (Figure 3). CNN classification performance is similar to baseline logistic regression models across the domain in terms of AUC, but outperforms logistic regression when using precision as the benchmark at ∼69% of grid cells. Our CNNs therefore offer a more conservative approach to parameterizing CG lightning as the false alarm rate, or CG lightning overprediction, is minimized compared to logistic regression in the majority of the WUS.

We use LRP_*z*_, an XAI technique, to investigate the regional relevance of predictor variables to successful CG lightning prediction. Using maximum LRP_*z*_ values, our results show that MSE_*ratio*_ and Γ_700‐500_ are consistently most relevant, and together represent the top CG lightning predictors for 96% of CNNs (Figure 4). These results are largely consistent when using LRP_*comp*_ (∼93%; Figure 5), and suggest an important role for relatively simple convective proxies when predicting the occurrence of CG lightning on daily timescales. We acknowledge that other variables that are physically important for lightning might not be prioritized by the CNNs during training (leading to lower relevance), likely due to variables such as MSE_*ratio*_ and Γ_700‐500_ offering more direct proxies for convection. Additionally, XAI techniques can struggle to produce physically truthful explanations when multicollinearity between predictors is present (Au et al., 2022; Krell et al., 2023).

To test the ability of the locally trained CNNs to reliably parameterize CG lightning occurrence across the WUS, we apply the trained models to meteorological fields over the full 1995–2022 period and generate CG lightning‐day predictions. Although there is general underprediction along the northern and western periphery and overprediction elsewhere, the differences between predicted and observed CG lightning days are within 25% for all CNNs (Figure 7b). Strong interannual correlation (domain median *r* = 0.87) between observed and predicted CG lightning days demonstrates that the CNNs also realistically capture the year‐to‐year variation of CG lightning days across the WUS (Figure 7c). These results lend confidence toward application of the CNNs to output from climate model projections, as they indicate that future lightning days can be reliably aggregated on seasonal timescales for comparison with the present climate. Additionally, we show that higher prediction confidence on individual days leads to more accurate predictions and correlates with increased CG lightning activity (Figure 8).

We note several possible limitations to our analysis. First, the relatively short CG lightning data record limits the sample size for training CNNs, particularly in western parts of the domain where lightning is rare. Second, the predictor variables used herein are spatially averaged over 1 degree latitude‐longitude and to a daily resolution, which could mask conditions favorable for CG lightning activity on finer spatial and sub‐daily timescales. In particular, this could help explain the lower classification performance in Pacific coastal states where lightning depends on more transient episodes of favorable meteorological conditions compared to areas in the monsoon core. Alternately, it is also possible that conditions may appear conducive to CG lightning at the daily resolution, but the necessary combination of ingredients may not align at the sub‐daily scale to produce thunderstorms. Third, constraints of the NLDN sensor network and the MERRA‐2 reanalysis could affect the identification of CG lightning and non‐lightning days and the CNN training based on the associated meteorological conditions. For instance, some CG lightning may be either missed or misclassified as cloud‐to‐cloud or intracloud lightning by the NLDN. Further, atmospheric reanalysis products may not always reliably simulate convective parameters compared to sounding observations (Taszarek et al., 2018).

Our manuscript makes a novel contribution to predicting CG lightning by using CNNs compared to existing parameterization efforts that use logistic regression or random forests, since CNNs explicitly incorporate spatial information of predictor variables. The CNNs are therefore able to capture spatial patterns of input variables that may hold predictive clues beyond their local values at that grid cell. Our methodology could be extended to predict days at higher thresholds of lightning activity or days with dry lightning (occurring with <2.5 mm daily precipitation). An important application of our CNN‐based parameterizations is to explore their use in GCMs to evaluate potential changes in CG lightning under different climates, a challenging problem that currently has high uncertainty since lightning is not directly simulated by GCMs. Our parameterizations are based on variables that are simulated by GCMs and avoid using cloud, precipitation, and convective mass variables that need to be parameterized in GCMs and can have large uncertainties (Etten‐Bohm et al., 2021). The locally trained CNNs developed in this study appear to be substantially more skillful at predicting CG lightning over the WUS compared to recent, large‐scale lightning parameterizations over the tropics and subtropics (e.g., Etten‐Bohm et al., 2021; Liu et al., 2022), highlighting the advantages of localized parameterizations. Applying the parameterizations developed in this study to GCMs would enable the quantification of future CG lightning activity across the WUS and refine projections of lightning‐caused wildfires across this region. Further, our approach of selecting suitable meteorological predictors and training local CNNs to parameterize lightning can be applied to other regions globally.

## Supporting information

Supporting Information S1

## Data Availability

National Lightning Detection Network data are sourced from the National Centers for Environmental Information Severe Weather Data Inventory (https://www.ncei.noaa.gov/pub/data/swdi/database‐csv/v2/). MERRA‐2 data were acquired from NASA's Goddard Earth Sciences Data Information Services Center (GES DISC; https://disc.gsfc.nasa.gov/datasets?keywords=merra‐2&page=1). Data sets used to perform analyses are available at the following Zenodo repository: https://doi.org/10.5281/zenodo.10685571 (Kalashnikov, 2024a). Analysis code can be accessed at the following Zenodo repository: https://doi.org/10.5281/zenodo.13905181 (Kalashnikov, 2024b).
