# Supplementary material for: Predicting Cloud‐To‐Ground Lightning in the Western United States From the Large‐Scale Environment Using Explainable Neural Networks
Source: J Geophys Res Atmos. 2024 Nov 22;129(22):e2024JD042147. doi: 10.1029/2024JD042147 (PMC11583119; doi:10.1029/2024JD042147)
Supplement: Supplementary file 1 — Supporting Information S1 [file JGRD-129-0-s001.pdf]

Journal of Geophysical Research: Atmospheres

Supporting Information for

Predicting Cloud-to-Ground Lightning in the Western United States from the Large-Scale Environment using Explainable Neural Networks

Dmitri A. Kalashnikov[1], Frances V. Davenport[2], Zachary M. Labe[3], Paul C. Loikith[4], John T. Abatzoglou[5], Deepti Singh[1]

[1]School of the Environment, Washington State University, Vancouver, WA, USA

[2]Civil and Environmental Engineering, Colorado State University, Fort Collins, CO, USA

[3]Atmospheric and Oceanic Sciences Program, Princeton University, Princeton, NJ, USA

[4]Department of Geography, Portland State University, Portland, OR, USA

[5]Management of Complex Systems Department, University of California, Merced, Merced, CA, USA

Contents of this file

Text S1 to S3
- S1: Column-integrated relative humidity
- S2: MSE ratio
- S3: Hyperparameter tuning

Tables S1 to S2
Figures S1 to S6

Text S1: *Column-integrated relative humidity*

The column-integrated relative humidity (CRH) is computed as follows:

$$\mathrm{IWV} = \frac{1}{g}\int_{p_t}^{p_b} q\,\mathrm{d}p \tag{1}$$

$$\mathrm{IWV}_s(p_t) = \frac{1}{g}\int_{p_t}^{p_b} q_s\,\mathrm{d}p \tag{2}$$

$$\mathrm{CRH} = \frac{\mathrm{IWV}}{\mathrm{IWV}_s} \tag{3}$$

where IWV and IWV_s are the integrated water vapor and its saturation counterpart, g is gravitational acceleration, q and q_s are the specific humidity and its saturation counterpart, and p_b and p_t are the pressures at the bottom (surface) and top of the air column, respectively. We set p_t = 300 hPa as atmospheric water content above this level is negligible (Mo et al., 2021).

Text S2: *MSE ratio*

The MSE_{ratio} is computed as follows:

$$\mathrm{MSE}_{surf} = c_pT + L_vq + gZ_s \tag{4}$$

$$\mathrm{MSE}_{sat} = c_pT_{500} + L_vqs_{500} + gZ_{500} \tag{5}$$

$$\mathrm{MSE}_{ratio} = \frac{\mathrm{MSE}_{surf}}{\mathrm{MSE}_{sat}} \tag{6}$$

where MSE_{surf} is the surface MSE, MSE_{sat} is the saturation MSE at 500 hPa, c_p is the specific heat of air at constant pressure, L_v is the latent heat of vaporization, g is the gravitational acceleration, T and T_{500} are the temperatures at 2 meters and 500 hPa, respectively, q is the specific humidity at 2 meters, qs_{500} is the saturation specific humidity at 500 hPa, and Z_s and Z_{500} are the geopotential heights at the surface and 500 hPa, respectively. We use the 500 hPa pressure level to represent the free troposphere following previous studies (Noyelle et al., 2023; Zhang & Boos, 2023).

Text S3: *Hyperparameter tuning*

For hyperparameter tuning, we implement a modified version of the hierarchical hyperparameter selection approach of Davenport & Diffenbaugh (2021). For each CNN, hyperparameters are sequentially tuned in pairs using a grid search that tests all parameter combinations for that pair, and for each combination we use 4-fold cross validation to

evaluate model performance (Davenport & Diffenbaugh, 2021). We first tune architecture parameters including the number of convolutional filters and the number of dense layers. Next, we tune the regularization parameters consisting of the L2 ("ridge") regularization factor and the dropout rate. Ridge regularization adds a penalty term to the loss function equivalent to the square of the neuron activations, thereby helping to reduce overfitting during training and improve model generalization (Belkin et al., 2019; Dagon et al., 2020; Davenport & Diffenbaugh, 2021). Dropout is a form of regularization that also helps limit overfitting by ignoring ("dropping out") a portion of neurons and their connections during training (Srivastava et al., 2014). Next, we tune the algorithm parameters consisting of the learning rate and batch size. Finally, we adjust the class weights that are applied during training. Class weights are helpful to address the class imbalance between CG lightning and non-lightning days at most grid cells, as CNNs could achieve high accuracy by simply predicting the minority class 100% of the time. For the minority class, weights are tested on a gradient from no class weight to the inverse of the class imbalance (Labe & Barnes, 2022).

Variable name	Variable abbreviation	Units
500 hPa geopotential height	Z_{500}	Standardized anomalies (σ)
500 hPa vertical velocity (Omega)	ω_{500}	Pa s^{-1}
700-500 hPa lapse rate	$\Gamma_{700\text{-}500}$	°C
Most unstable convective available potential energy	MUCAPE	J kg^{-1}
Column-integrated relative humidity	CRH	%
500 hPa relative humidity	RH_{500}	%
Ratio of surface moist static energy (MSE) to free-troposphere saturation MSE	MSE_{ratio}	Unitless

Table S1. Meteorological variables used predict the occurrence of CG lightning.

Hyperparameter	Values tested
Number of convolutional filters	[8,16,32]
Number of dense layers	[1,2,3]
L2 “ridge” regularization	[0.00001,0.0001,0.001,0.01,0.1,0.5,1]
Dropout rate	[0.1,0.2,0.3,0.4,0.5]
Learning rate	[0.00001,0.0001,0.001,0.01,0.1,0.5,1]
Batch size	[32,64,128,256,512,1024]
Class weights	10 equally spaced increments between 1 and the inverse of class imbalance

Table S2. Tuned hyperparameters and the range of values over which optimization is conducted.

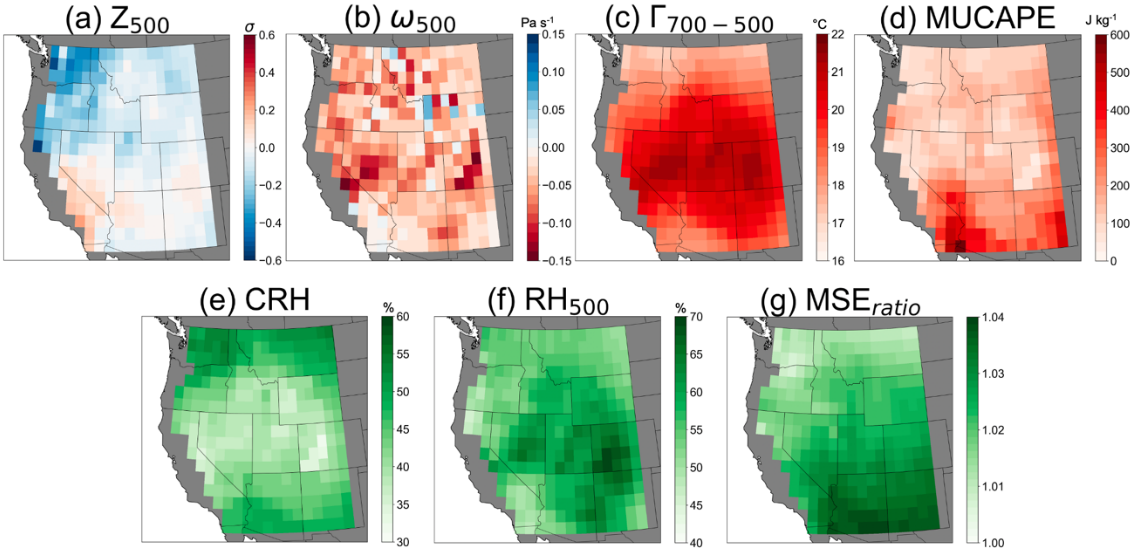

Figure S1. Composites of meteorological variables on CG lightning days at each 1° x 1° grid cell during June-September, 1995-2022. Differences from non-lightning days are shown in Figure 1.

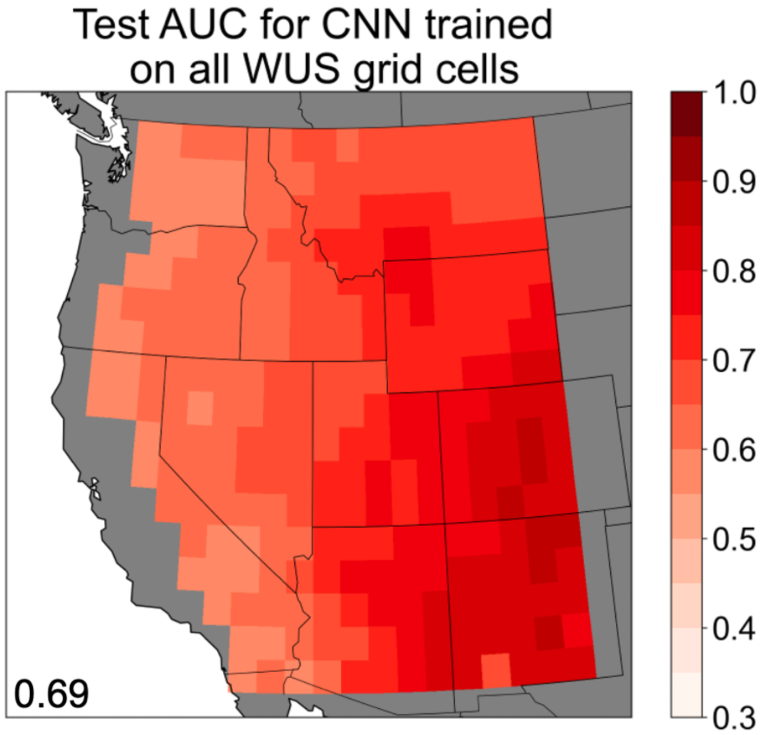

Figure S2. Precision-recall AUC for CNN trained on all WUS grid cells. Inset value shows domain-median AUC.

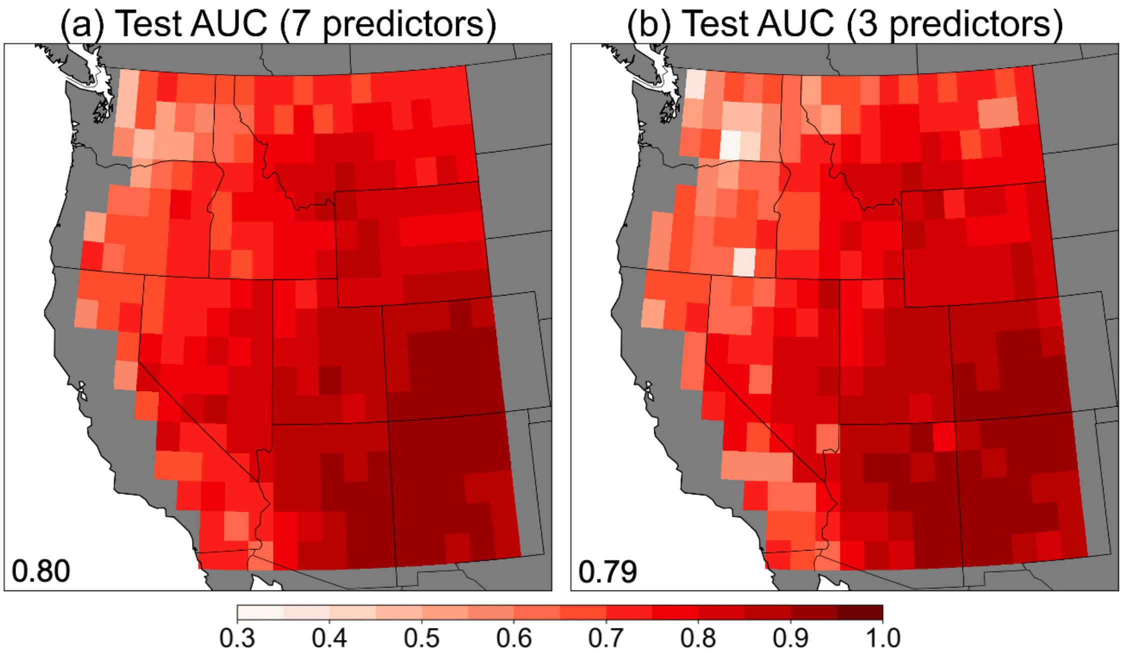

Figure S3. Comparison of precision-recall AUC between (a) the full 7-predictor CNNs and (b) reduced CNNs with 3 predictor variables (MSE_{ratio}, $\Gamma_{700\text{-}500}$, RH_{500}). Inset values show domain-median AUC.

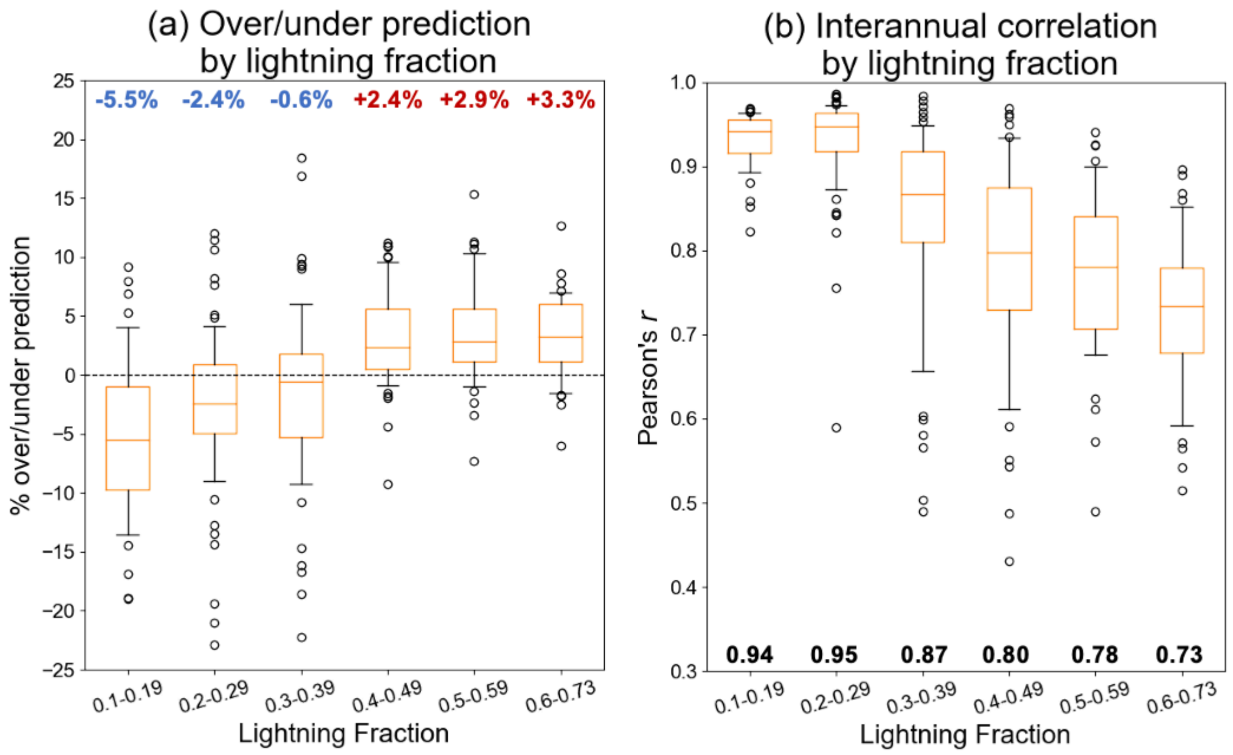

Figure S4. (a) CNN prediction error by lightning fraction with median values for each fraction bin shown above boxplots. Data corresponds to map in Figure 7b. (b) Interannual correlation by lightning fraction with median values for each fraction bin shown below boxplots. Data corresponds to map in Figure 7c.

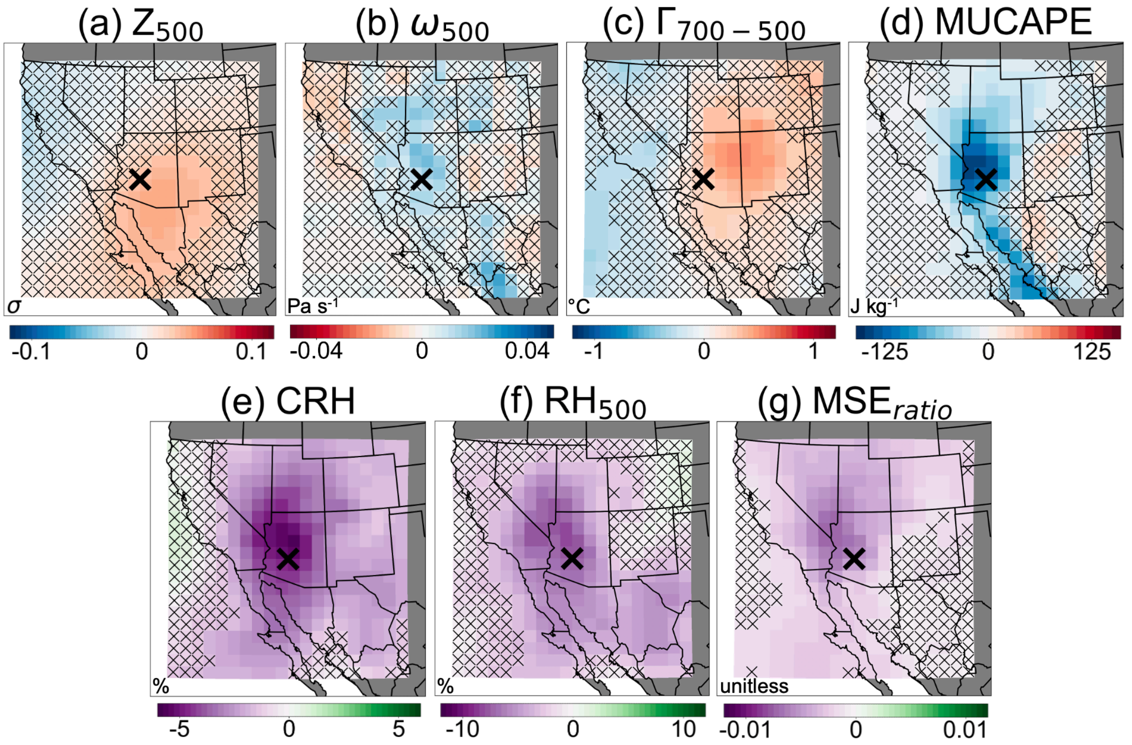

Figure S5. Difference in meteorological variables between false positive days (when CG lightning was predicted but did not occur) and true positive days (when CG lightning was correctly predicted) for the CNN with the largest overprediction in the domain (black marker). This grid cell is located in Arizona (33.5°N, 112.5°W) and corresponds to Cell 4 in Figure 7. Positive values indicate higher quantity on false positive days (false positive minus true positive). Hatching indicates that differences are insignificant ($p \geq 0.05$) between false positive and true positive days according to a two-tailed *t*-test.

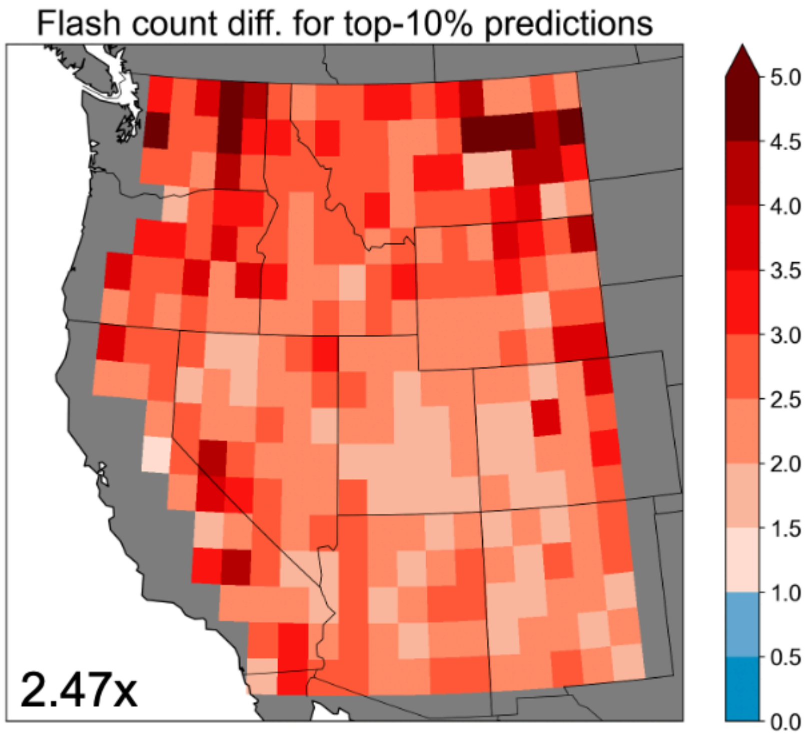

Figure S6. Enhancement of CG lightning flash counts for the top-10% highest probability CG lightning-day predictions at each grid cell, versus all other CG lightning-day predictions. Inset text shows domain-median enhancement.
